# Supplementary material for: NFTsim: Theory and Simulation of Multiscale Neural Field Dynamics
Source: PLoS Comput Biol. 2018 Aug 22;14(8):e1006387. doi: 10.1371/journal.pcbi.1006387 (PMC6122812; doi:10.1371/journal.pcbi.1006387)
Supplement: S1 Appendix — (PDF) [file pcbi.1006387.s001.pdf]

S1 Appendix. Discretization of the wave equation.

In this section we describe the discretization of the wave equation. This method allows us to obtain an equation to advance each field ϕ_{ab} from t to $t + \Delta t$. We remind the reader that the equation relating the field $\phi_{ab}(\mathbf{r}, t)$ to the driving signal $Q_b(\mathbf{r}, t)$ is

$$\left[\frac{1}{\gamma_{ab}^2}\frac{\partial^2}{\partial t^2} + \frac{2}{\gamma_{ab}}\frac{\partial}{\partial t} + 1 - r_{ab}^2\nabla^2\right]\phi_{ab}(\mathbf{r}, t) = Q_b(\mathbf{r}, t). \tag{1}$$

This equation is a damped wave equation for $\phi_{ab}(\mathbf{r}, t)$ with source $Q_b(\mathbf{r}, t)$. The damping is introduced via the first-order derivative term in the same way friction forces enter a vibrating mechanical system; and, by the third term in Eq. (1). This equation can be simplified by making the following substitutions

$$u = \phi_{ab}\exp(\gamma_{ab}t), \tag{2}$$

and

$$w = Q_b\exp(\gamma_{ab}t). \tag{3}$$

We then obtain the undamped wave equation

$$\left[\frac{1}{\gamma_{ab}^2}\frac{\partial^2}{\partial t^2} - r_{ab}^2\nabla^2\right]u(\mathbf{r}, t) = w(\mathbf{r}, t), \tag{4}$$

To solve this differential equation numerically, we replace the temporal and spatial derivatives with finite central difference approximations on a discretized domain. The derivation presented in the following paragraphs solves the Eq. (4) by using explicit methods, that is, the next value of ϕ_{ab} is computed from known past values of u and w and all future time terms appear on the same side of the time stepping equation.

Consider first the term $\partial^2/\partial t^2$ in Eq. (4), and let the superscripts n index time in units of $k = \Delta t$. We can use a Taylor expansion to write

$$\delta_t^2 u^n = u^{n+1} - 2u^n + u^{n-1}, \tag{5}$$

$$= 2\left(\frac{k^2}{2!}\frac{\partial^2 u}{\partial t^2} + \frac{k^4}{4!}\frac{\partial^4 u}{\partial t^4} + ...\right), \tag{6}$$

$$= k^2\left(\frac{\partial^2 u}{\partial t^2} + \frac{k^2}{12}\frac{\partial^4 u}{\partial t^4} + ...\right), \tag{7}$$

where δ_t^2 is the second order central difference operator in time; and, u^{n+1} is the future term we are interested in calculating. Combining Eqs. (5) and (7) yields

$$u^{n+1} = 2u^n - u^{n-1} + k^2\left(\frac{\partial^2 u}{\partial t^2} + \frac{k^2}{12}\frac{\partial^4 u}{\partial t^4} + ...\right). \tag{8}$$

Note that this approximation is $\mathcal{O}(k^2)$ accurate in time because we use a second order central difference formula to approximate the second order derivative. So, the error is proportional to the square of k. In a similar way, the second order centered finite difference approximation for the second order spatial derivatives are

$$\delta_{x_1}^2 u_{l,m} \equiv u_{l+1,m} - 2u_{l,m} + u_{l-1,m} = h^2\left(\frac{\partial^2 u}{\partial x_1^2} + \frac{h^2}{12}\frac{\partial^4 u}{\partial x_1^4} + ...\right), \tag{9}$$

$$\delta_{x_2}^2 u_{l,m} \equiv u_{l,m+1} - 2u_{l,m} + u_{l,m-1} = h^2\left(\frac{\partial^2 u}{\partial x_2^2} + \frac{h^2}{12}\frac{\partial^4 u}{\partial x_2^4} + ...\right), \tag{10}$$

where $h = \Delta x_1 = \Delta x_2$ is the grid spacing and the subscripts m and l index grid points in the orthogonal x_1 and x_2 directions, respectively. The error of the centered difference scheme used here is $\mathcal{O}(h^2)$. We also use:

$$\nabla^2 = \frac{\partial^2}{\partial x_1^2} + \frac{\partial^2}{\partial x_2^2}, \tag{11}$$

$$= \frac{1}{h^2}(\delta_{x_1}^2 + \delta_{x_2}^2), \tag{12}$$

and

$$\frac{\partial^2 u}{\partial t^2} = \gamma_{ab}^2 r_{ab}^2 \nabla^2 u + \gamma_{ab}^2 w, \tag{13}$$

$$\frac{\partial^4 u}{\partial t^4} = \gamma_{ab}^2 r_{ab}^2 \nabla^2 \frac{\partial^2 u}{\partial t^2} + \gamma_{ab}^2 \frac{\partial^2 w}{\partial t^2}. \tag{14}$$

We then substitute Eqs (13) and (14) into Eq. (8) and obtain

$$\begin{aligned} u^{n+1} &= 2u^n - u^{n-1} \\ &+ k^2 \left[\gamma_{ab}^2 r_{ab}^2 \nabla^2 u + \gamma_{ab}^2 w + \frac{k^2}{12} \left(\gamma_{ab}^2 r_{ab}^2 \nabla^2 \left(\frac{\partial^2 u}{\partial t^2} \right) + \gamma_{ab}^2 \frac{\partial^2 w}{\partial t^2} \right) \right], \end{aligned} \tag{15}$$

and further substitute the term $\frac{\partial^2 u}{\partial t^2}$ in Eq. (15) for the right hand side of Eq. (13)

$$\begin{aligned} u^{n+1} &= 2u^n - u^{n-1} \\ &+ k^2 \left[\gamma_{ab}^2 r_{ab}^2 \nabla^2 u + \gamma_{ab}^2 w + \frac{k^2}{12} \left(\gamma_{ab}^2 r_{ab}^2 \nabla^2 \left(\gamma_{ab}^2 r_{ab}^2 \nabla^2 u + \gamma_{ab}^2 w \right) + \gamma_{ab}^2 \frac{\partial^2 w}{\partial t^2} \right) \right]. \end{aligned} \tag{16}$$

By rearranging the terms in Eq. (16) we can express u^{n+1} in terms of u and w

$$\begin{aligned} u^{n+1} &= 2u^n - u^{n-1} \\ &+ k^2 \left[\gamma_{ab}^2 r_{ab}^2 \nabla^2 u + \gamma_{ab}^2 w + \frac{k^2}{12} \left(\gamma_{ab}^4 r_{ab}^4 \nabla^4 u + \gamma_{ab}^4 r_{ab}^2 \nabla^2 w + \gamma_{ab}^2 \frac{\partial^2 w}{\partial t^2} \right) \right], \end{aligned} \tag{17}$$

$$\begin{aligned} u^{n+1} &= 2u^n - u^{n-1} + k^2 \gamma_{ab}^2 r_{ab}^2 \left[\nabla^2 + \frac{k^2 \gamma_{ab}^2 r_{ab}^2}{12} \nabla^4 \right] u \\ &+ k^2 \gamma_{ab}^2 \left[1 + \frac{k^2}{12} \frac{\partial^2}{\partial t^2} + \frac{k^2 \gamma_{ab}^2 r_{ab}^2}{12} \nabla^2 \right] w. \end{aligned} \tag{18}$$

We now omit the terms involving ∇^4 since a second order approximation is enough, giving

$$u^{n+1} = 2u^n - u^{n-1} + k^2 \gamma_{ab}^2 r_{ab}^2 \nabla^2 u^n + k^2 \gamma_{ab}^2 \left[1 + \frac{k^2}{12} \frac{\partial^2}{\partial t^2} + \frac{k^2 \gamma_{ab}^2 r_{ab}^2}{12} \nabla^2 \right] w. \tag{19}$$

Next we replace ∇^2 by the approximations defined in Eq. (12) to obtain

$$\begin{aligned} u^{n+1} &= 2u^n - u^{n-1} \\ &+ p^2 (\delta_{x_1}^2 + \delta_{x_2}^2) u^n + k^2 \gamma_{ab}^2 \left[1 + \frac{1}{12} \delta_t^2 + \frac{p^2}{12} \left(\delta_{x_1}^2 + \delta_{x_2}^2 \right) \right] w, \end{aligned} \tag{20}$$

where $p \equiv p_{ab} = k\gamma_{ab}r_{ab}/h$ is the Courant number. Next, we replace the second order difference operators δ_t^2, $\delta_{x_1}^2$, and $\delta_{x_2}^2$ to obtain an explicit solution to compute the next value in time of $u_{m,l}$:

$$u_{m,l}^{n+1} = 2u_{m,l}^{n} - u_{m,l}^{n-1} + p^2(\delta_{x_1}^2 + \delta_{x_2}^2)u_{m,l}^{n} + k^2\gamma_{ab}^2\left[1 + \frac{1}{12}\delta_t^2 + \frac{p^2}{12}\left(\delta_{x_1}^2 + \delta_{x_2}^2\right)\right]w_{m,l}^{n}, \tag{21}$$

$$\begin{aligned} u_{m,l}^{n+1} &= 2u_{m,l}^{n} - u_{m,l}^{n-1} + p^2(u_{m,l+1}^{n} + u_{m,l-1}^{n} + u_{m+1,l}^{n} + u_{m-1,l}^{n} - 4u_{m,l}^{n}) \\ &+ k^2\gamma_{ab}^2\left[w_{m,l}^{n} + \frac{1}{12}(w_{m,l}^{n+1} - 2w_{m,l}^{n} + w_{m,l}^{n-1})\right. \\ &\left.+\frac{p^2}{12}\left(w_{m,l+1}^{n} + w_{m,l-1}^{n} + w_{m+1,l}^{n} + w_{m-1,l}^{n} - 4w_{m,l}^{n}\right)\right], \end{aligned} \tag{22}$$

$$\begin{aligned} u_{m,l}^{n+1} &= 2u_{m,l}^{n} - 4p^2u_{m,l}^{n} + p^2(u_{m,l+1}^{n} + u_{m,l-1}^{n} + u_{m+1,l}^{n} + u_{m-1,l}^{n}) - u_{m,l}^{n-1} \\ &+ \frac{k^2\gamma_{ab}^2}{12}\left[12w_{m,l}^{n} + w_{m,l}^{n+1} - 2w_{m,l}^{n} + w_{m,l}^{n-1}\right. \\ &\left.+p^2\left(w_{m,l+1}^{n} + w_{m,l-1}^{n} + w_{m+1,l}^{n} + w_{m-1,l}^{n} - 4w_{m,l}^{n}\right)\right], \end{aligned} \tag{23}$$

$$\begin{aligned} u_{m,l}^{n+1} &= (2 - 4p^2)u_{m,l}^{n} + p^2(u_{m,l+1}^{n} + u_{m,l-1}^{n} + u_{m+1,l}^{n} + u_{m-1,l}^{n}) - u_{m,l}^{n-1} \\ &+ \frac{k^2\gamma_{ab}^2}{12}\left[(10 - 4p^2)w_{m,l}^{n} + (w_{m,l}^{n+1} + w_{m,l}^{n-1})\right. \\ &\left.+p^2\left(w_{m,l+1}^{n} + w_{m,l-1}^{n} + w_{m+1,l}^{n} + w_{m-1,l}^{n}\right)\right]. \end{aligned} \tag{24}$$

From Eqs (2) and (3), $u^n = \phi^n e^{\gamma_{ab} n\Delta t}$ and $w^n = \phi^n e^{\gamma_{ab} n\Delta t}$. Also, for a single simulation step, the current state is centered at $t = 0$ and thus indexed by $n = 0$; the next and previous states are ± 1 step away, or equivalently $\pm\Delta t$. Then, $n + 1$ denotes time Δt and $n - 1$ denotes time $-\Delta t$. Therefore we define the following substitutions

$$u^{n+1} = \phi^{n+1}e^{\gamma_{ab}\Delta t}, \tag{25}$$

$$u^{n} = \phi^{n}, \tag{26}$$

$$u^{n-1} = \phi^{n-1}e^{-\gamma_{ab}\Delta t}, \tag{27}$$

$$w^{n+1} = Q^{n+1}e^{\gamma_{ab}\Delta t}, \tag{28}$$

$$w^{n} = Q^{n}, \tag{29}$$

$$w^{n-1} = Q^{n-1}e^{-\gamma_{ab}\Delta t}, \tag{30}$$

The spatial indices are omitted for compactness but can take the values $\{m, m \pm 1\}$ and $\{l, l \pm 1\}$. Hence, Eq. (24) can be expressed in terms of ϕ and Q as

$$\begin{aligned} \phi_{m,l}^{n+1}e^{\gamma_{ab}\Delta t} &= (2 - 4p^2)\phi_{m,l}^{n} + p^2(\phi_{m,l+1}^{n} + \phi_{m,l-1}^{n} + \phi_{m+1,l}^{n} + \phi_{m-1,l}^{n}) - \phi_{m,l}^{n-1}e^{-\gamma_{ab}\Delta t} \\ &+ \frac{k^2\gamma_{ab}^2}{12}\Big[(10 - 4p^2)Q_{m,l}^{n} + (Q_{m,l}^{n+1}e^{\gamma_{ab}\Delta t} + Q_{m,l}^{n-1}e^{-\gamma_{ab}\Delta t}) \\ &+p^2\left(Q_{m,l+1}^{n} + Q_{m,l-1}^{n} + Q_{m+1,l}^{n} + Q_{m-1,l}^{n}\right)\Big]. \end{aligned} \tag{31}$$

Finally, upon multiplying both sides of Eq. (31) by $e^{-\gamma_{ab}\Delta t}$ one finds

$$\phi_{m,l}^{n+1} = e^{-\gamma_{ab}\Delta t}\Big\{(2-4p^2)\phi_{m,l}^{n} + p^2(\phi_{m,l+1}^{n} + \phi_{m,l-1}^{n} + \phi_{m+1,l}^{n} + \phi_{m-1,l}^{n}) - \phi_{m,l}^{n-1}e^{-\gamma_{ab}\Delta t}$$
$$+ \frac{k^2\gamma_{ab}^2}{12}\Big[(10-4p^2)Q_{m,l}^{n} + (Q_{m,l}^{n+1}e^{\gamma_{ab}\Delta t} + Q_{m,l}^{n-1}e^{-\gamma_{ab}\Delta t})$$
$$+ p^2\left(Q_{m,l+1}^{n} + Q_{m,l-1}^{n} + Q_{m+1,l}^{n} + Q_{m-1,l}^{n}\right)\Big]\Big\}. \tag{32}$$

Eq. (32) is the formula to advance an axonal field ϕ_{ab} one time step based on its current state (n) and previous state ($n-1$) when ϕ_{ab} is governed by Eq. (1).
