# Supplementary material for: NFTsim: Theory and Simulation of Multiscale Neural Field Dynamics
Source: PLoS Comput Biol. 2018 Aug 22;14(8):e1006387. doi: 10.1371/journal.pcbi.1006387 (PMC6122812; doi:10.1371/journal.pcbi.1006387)
Supplement: S2 Appendix — (PDF) [file pcbi.1006387.s002.pdf]

S2 Appendix. Configuration file used in Analysis and Visualization.

```
e-erps-all-nodes.conf - configuration file for one-population neural field model.
All parameters are in SI units.

Time: 0.25 Deltat: 2.44140625e-4
Nodes: 4096

    Connection matrix:
From:  1  2
To 1:  1  2
To 2:  0  0

Population 1: Excitatory
Length: 0.5
Q: 10
Firing: Function: Sigmoid Theta: 0.01292 Sigma: 0.0038 Qmax: 340
 Dendrite 1: alpha: 83 beta: 769
 Dendrite 2: alpha: 83 beta: 769

Population 2: Stimulation
Length: 0.5
 Stimulus: Superimpose: 2
   Stimulus: Pulse - Onset: 0.03125 Node: 2000 Amplitude:  2
                     Width: 0.001953125 Frequency: 1 Pulses: 1
   Stimulus: Pulse - Onset: 0.06250 Node: 2097 Amplitude: -2
                     Width: 0.001953125 Frequency: 1 Pulses: 1

Propagator 1: Wave - Tau: 0 Range: 0.2 gamma: 30
Propagator 2: Map -

Coupling 1:  Map - nu: 0
Coupling 2:  Map - nu: 1e-4

Output: Node: All Start: 0 Interval: 9.765625e-4
Population:
Dendrite:
Propagator: 1.phi
Coupling:
```
